# Supplementary figures and images for: From Plants to Ants: Fungal Modification of Leaf Lipids for Nutrition and Communication in the Leaf-Cutter Ant Fungal Garden Ecosystem
Source: mSystems. 2021 Mar 23;6(2):e01307-20. doi: 10.1128/mSystems.01307-20 (PMC8547007; doi:10.1128/mSystems.01307-20)

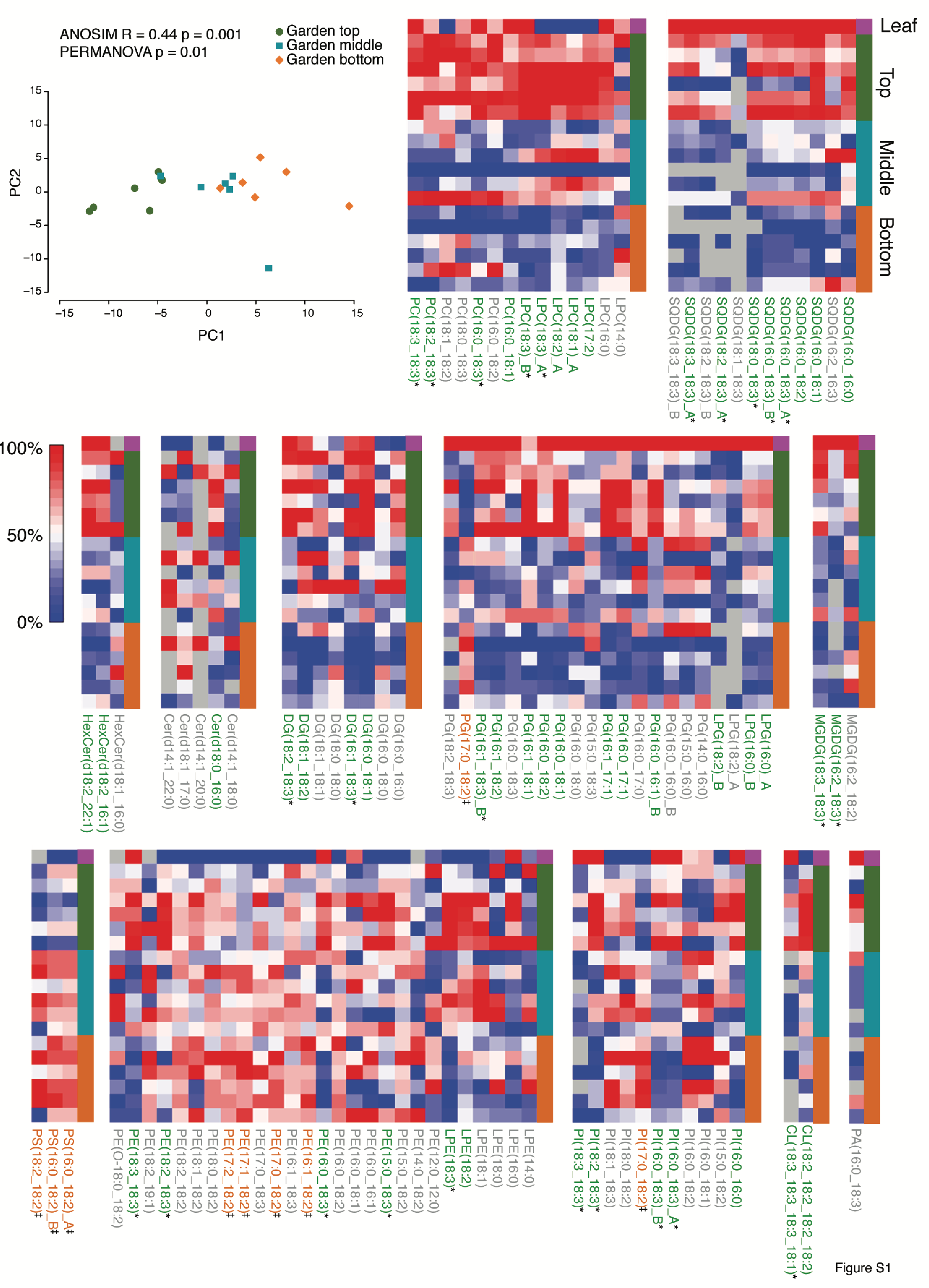

Supplement: FIG S1 [file msystems.01307-20-sf001.tif]

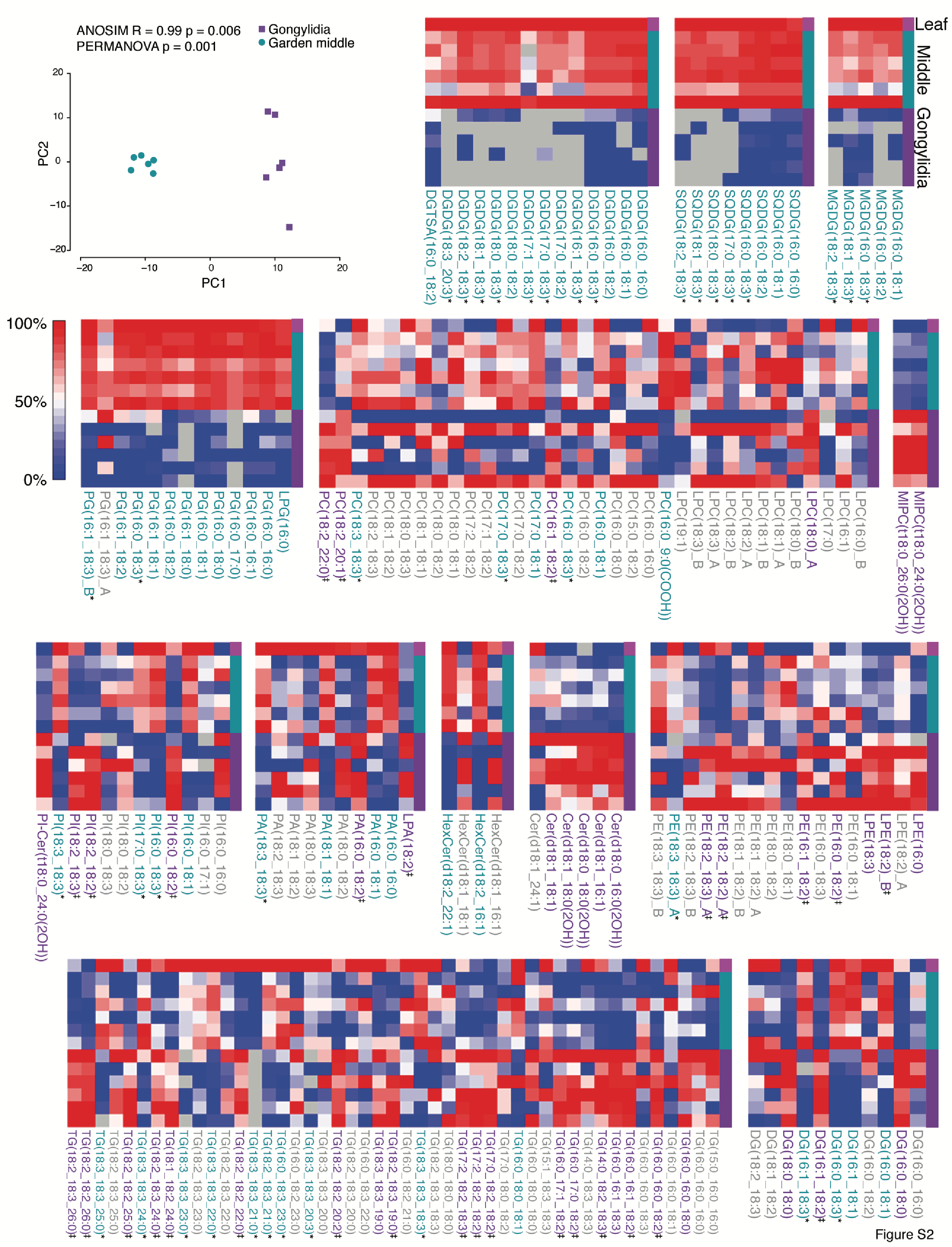

Supplement: FIG S2 [file msystems.01307-20-sf002.tif]

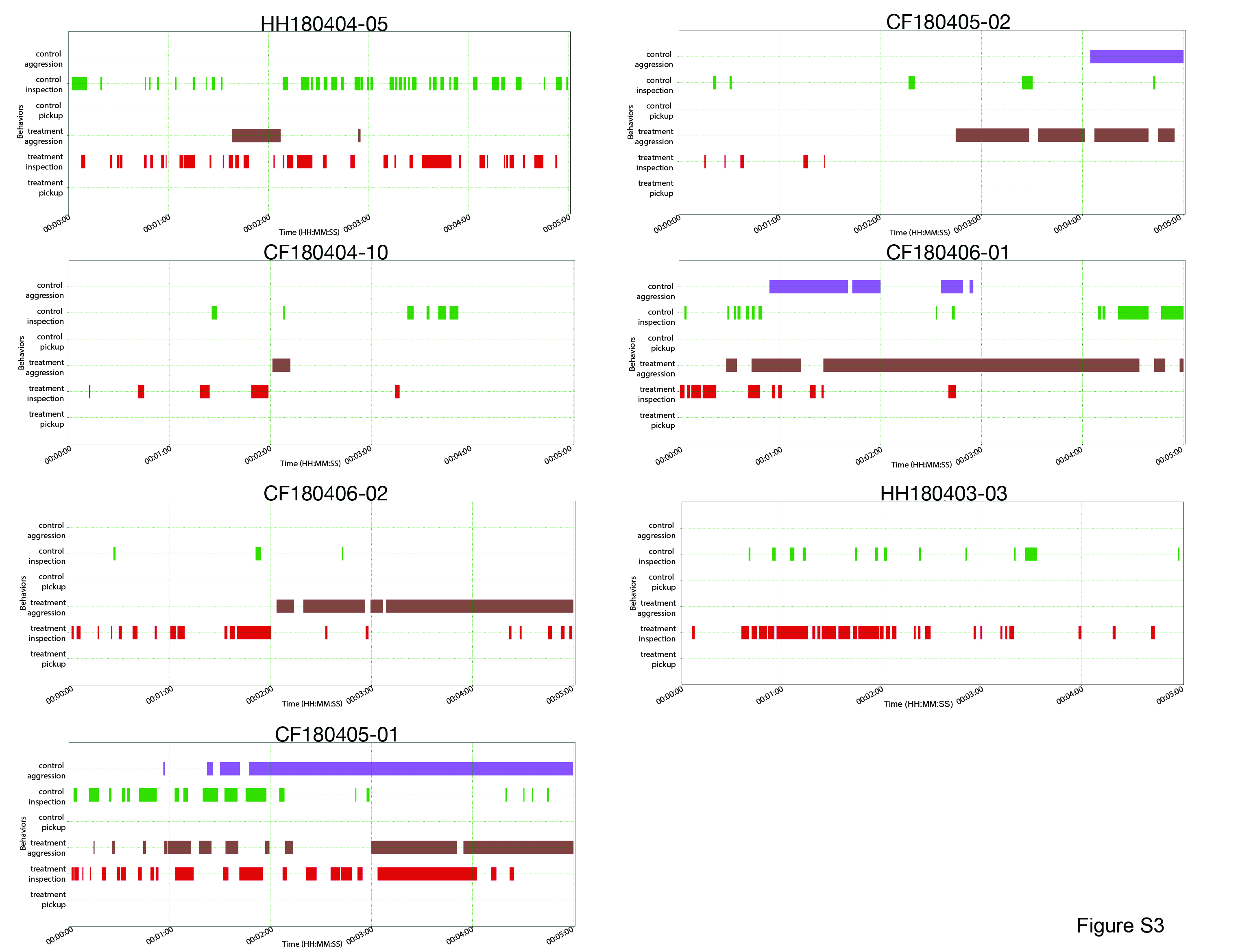

Supplement: FIG S3 [file msystems.01307-20-sf003.tif]

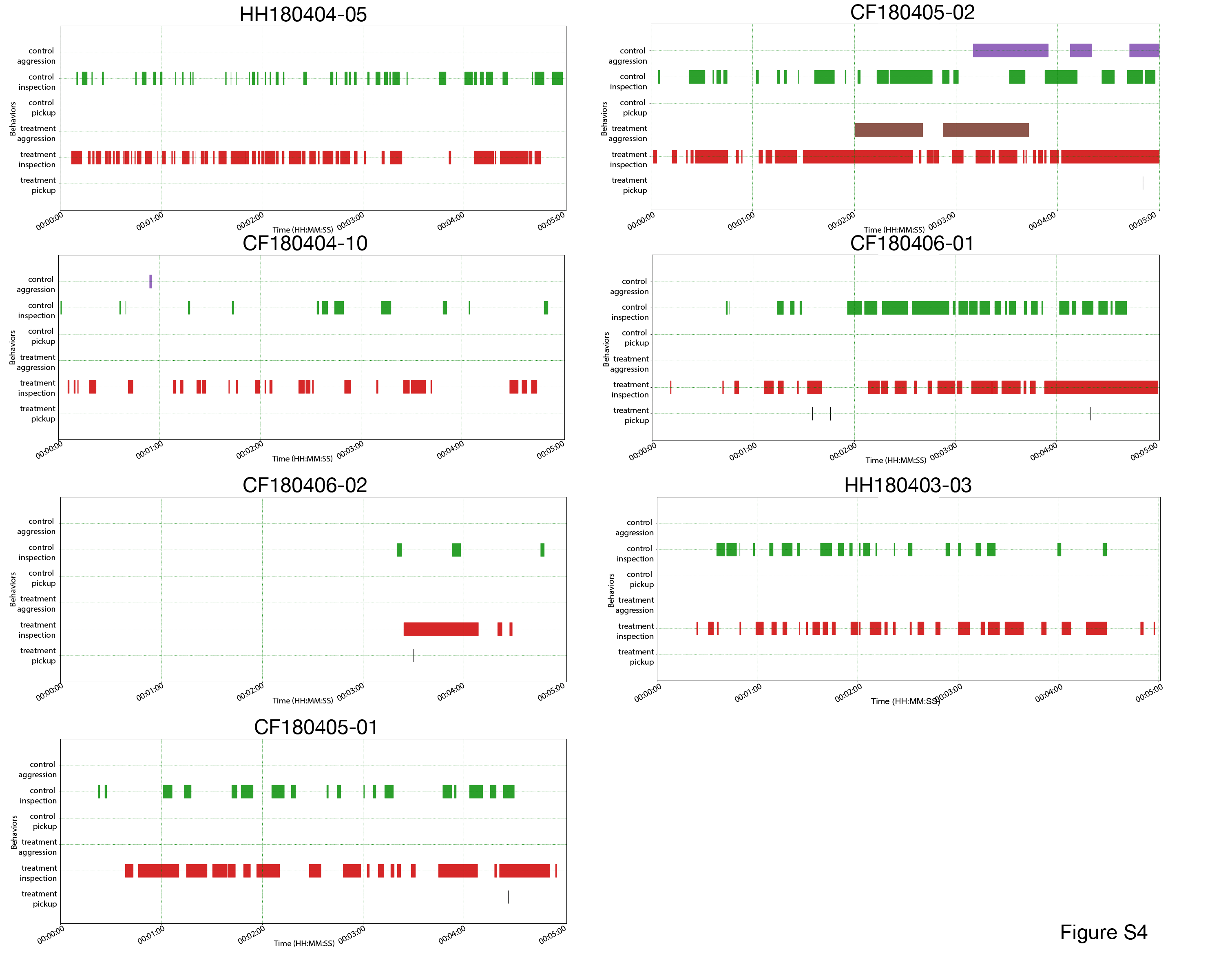

Supplement: FIG S4 [file msystems.01307-20-sf004.tif]

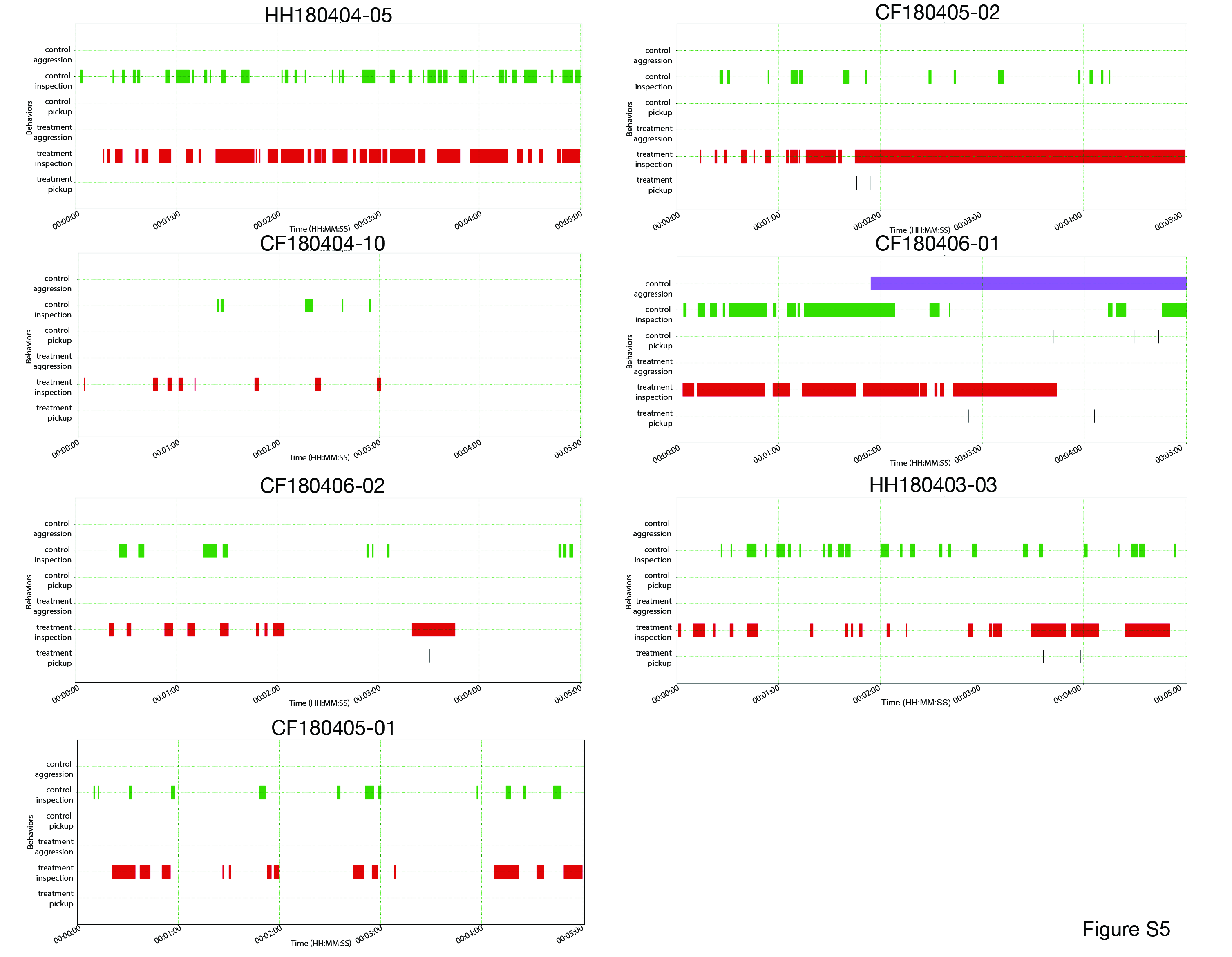

Supplement: FIG S5 [file msystems.01307-20-sf005.tif]
